# Melatonin Attenuates Potato Late Blight by Disrupting Cell Growth, Stress Tolerance, Fungicide Susceptibility and Homeostasis of Gene Expression in *Phytophthora infestans*

**DOI:** 10.3389/fpls.2017.01993

**Published:** 2017-11-21

**Authors:** Shumin Zhang, Xianzhe Zheng, Russel J. Reiter, Shun Feng, Ying Wang, Sen Liu, Liang Jin, Zhengguo Li, Raju Datla, Maozhi Ren

**Affiliations:** ^1^School of Life Sciences, Chongqing University, Chongqing, China; ^2^School of Basic Medical Sciences, North Sichuan Medical College, Nanchong, China; ^3^Department of Cellular and Structural Biology, University of Texas Health Science Center at San Antonio, San Antonio, TX, United States; ^4^College of Agronomy and Biotechnology, Southwest University, Chongqing, China; ^5^Plant Biotechnology Institute, National Research Council of Canada, Saskatoon, SK, Canada

**Keywords:** *Phytophthora infestans*, melatonin, potato late blight, stress tolerance, fungicide susceptibility, transcriptome

## Abstract

*Phytophthora infestans* (*P. infestans*) is the causal agent of potato late blight, which caused the devastating Irish Potato Famine during 1845-1852. Until now, potato late blight is still the most serious threat to potato growth and has caused significant economic losses worldwide. Melatonin can induce plant innate immunity against pathogen infection, but the direct effects of melatonin on plant pathogens are poorly understood. In this study, we investigated the direct effects of melatonin on *P. infestans*. Exogenous melatonin significantly attenuated the potato late blight by inhibiting mycelial growth, changing cell ultrastructure, and reducing stress tolerance of *P. infestans*. Notably, synergistic anti-fungal effects of melatonin with fungicides on *P. infestans* suggest that melatonin could reduce the dose levels and enhance the efficacy of fungicide against potato late blight. A transcriptome analysis was carried out to mine downstream genes whose expression levels were affected by melatonin. The analysis of the transcriptome suggests that 66 differentially expressed genes involved in amino acid metabolic processes were significantly affected by melatonin. Moreover, the differentially expressed genes associated with stress tolerance, fungicide resistance, and virulence were also affected. These findings contribute to a new understanding of the direct functions of the melatonin on *P. infestans* and provide a potential ecofriendly biocontrol approach using a melatonin-based paradigm and application to prevent potato late blight.

## Introduction

*Phytophthora infestans* is a notorious oomycete pathogen, as the causal agent of potato late blight, which triggered the Great Irish Famine in the mid-nineteenth century (Pearson, 2011). Potato late blight is the most serious threat to potato plants and causes significant economic losses worldwide (Haverkort et al., 2008). In recent decades, several chemical fungicides have been developed and applied for controlling potato late blight by targeting the typical mechanisms of impairing the respiratory chain, disrupting metabolic homeostasis and inhibiting RNA polymerase of *P. infestans* (Matheron and Porchas, 2000; Mitani et al., 2001; Gullino et al., 2010). However, the highly complex and huge genome of *P. infestans* confers this pathogen the capacity of rapid evolution to overcome these chemical fungicides by altering a single gene (Haas et al., 2009). Moreover, it is important that the environmental, health and safety concerns associated with the chemical fungicides be taken into consideration. Thus, the need to develop alternatives to fungicide-based approaches that offers less toxicity, more target-specific and environmental-sustainability is desired and preferred.

Melatonin, a natural product also referred as N-acetyl-5-methoxytryptamine, is widely present in animals, plants, and microbes (Arendt, 2005; Pandi-Perumal et al., 2006; Reiter et al., 2014). Initially, its function is known to regulate circadian rhythms. Subsequently, other functions including the modulation of mood, sleep, metabolism, and antioxidant are also established in diverse organisms (Reiter et al., 2009, 2015, 2016; Arnao and Hernandezruiz, 2015; Manchester et al., 2015). Specifically, melatonin has been reported to be effective in inhibiting the cell growth in some human pathogens by impairing their mitochondrial functions, inhibiting biofilm formation, and reducing intracellular substrates (Tekbas et al., 2008; Elmahallawy et al., 2014; Yang et al., 2014). For example, melatonin inhibits *Leishmania* via impairing the mitochondrial functions (Elmahallawy et al., 2014). Besides, melatonin mediated inhibition of the growth of a variety of cancer cells, such as hepatoma via the interference of fatty acid metabolism (Blask et al., 1999a,b; Sauer et al., 2001), colorectal cancer via the decrease of MT1 (melatonin receptor 1) (Farriol et al., 2000; Nemeth et al., 2011), pituitary tumor via the disturbance of nuclear receptor (Karasek et al., 2003). Importantly, a number of clinical trials have also confirmed that the use of melatonin is an effective measure to control a variety of disease, including the infectious diseases caused by pathogenic bacteria or virus, such as sepsis, Herpes (Sanchezbarcelo et al., 2010).

In plants, melatonin increases the resistance against pathogens via activating the expression of defense genes, elevating NO production, and thickening the cell wall (Yin et al., 2013; Lee et al., 2014, 2015; Qian et al., 2015; Shi et al., 2015, 2016; Zhao et al., 2015; Lee and Back, 2016; Wei et al., 2017). For example, various pathogenesis-related (PR) and defense genes that are activated by salicylic acid (SA) and ethylene (ET), have also been shown to be induced by melatonin in the Arabidopsis and tobacco (Lee et al., 2014, 2015). Melatonin also induces NO production, which plays an important role in plant innate immunity response against bacterial pathogen attacks (Shi et al., 2015). It is also observed that melatonin is effective for thickening cell wall by inducing the accumulation of cellulose, galactose, xylose, and callose in plants to prevent pathogen infection (Qian et al., 2015; Zhao et al., 2015). Recently, studies on melatonin against plant pathogens indirectly by triggering the plant immunity have been explored. However, little is known about the direct interaction between melatonin and plant pathogens (Arnao and Hernandezruiz, 2015).

In this study, we investigated the direct effects of melatonin on *P. infestans*. The results and observations showed that the mycelial growth, cell ultrastructure, stress tolerance, and fungicide susceptibility of *P. infestans* could be significantly altered in the presence of melatonin. Because of the lack of the potato varieties resistant to late blight, the control of this notorious disease largely depends on the high dosage and frequency of fungicide application. Interestingly, the dosage of fungicide can be significantly reduced when it was combined with melatonin, which would be important for human health and the environment. Majority of the previous transcriptome studies focused on the host-*P. infestans* interaction (Gao et al., 2013; Ali et al., 2014; Frades et al., 2015; Ah-Fong et al., 2017), but our analysis of transcriptome were focused to reveal the underlying mechanism of melatonin against *P. infestans*. The key findings of this study will be useful to explore the melatonin-based alternate approaches for the late blight control in potato.

## Materials and methods

### *P. infestans* strain, media, and culture conditions

T30-4 (A1 mating type) is the sequenced *P. infestans* isolate that is commonly used in laboratory research worldwide (Haas et al., 2009). It was provided by Dr. Suomeng Dong, Nanjing Agriculture University, China. The strain was cultured on Rye A agar at 18°C in the dark as described in previous report (Avrova et al., 2003).

### Effects of melatonin on late blight of potato infected by *P. infestans* T30-4

*P. infestans* strain T30-4 was cultured on Rye A agar at 18°C in the dark for 14 days (Avrova et al., 2003). Then, the potato leaves and the tuber slices (2 cm × 3 cm × 3 mm in size) were sprayed with melatonin solution in varying concentrations (0, 1, 3, 6, 8, 10 mM) dissolved in DMSO, and the same volumes of DMSO as the control, and the water also as the control (CK) (excluding the influence on leaves and tubers by melatonin or DMSO). Then, the 7-mm-diameter T30-4 mycelial disks were put on the potato tuber slices and leaves (the melatonin team and DMSO team), then incubated at 18°C with a 12-h/12-h light/dark cycle for 5 days. The size of each lesion was measured then analyzed with the Student's *t*-test. Three biological repeats were performed for each experiment.

### Measuring the effects of melatonin on mycelial growth of *P. infestans*

The 7-mm-diameter T30-4 mycelial disks were cultured on Rye A agar plates supplemented with varying concentrations of melatonin solution (0, 1, 1.5, 2, 3, and 5 mM), and the same volumes of DMSO as the control. The diameters of the colony were measured at the 9th day and 14th day. The inhibition rates were calculated using the diameter of control colony (D), drug-treated colony (M) as follows: [(D – M)/(D – 0.7)] × 100%. All experiments were repeated three times.

### Measurements of the effects of melatonin on cell viability of *P. infestans*

MTT, 3-(4, 5-dimethyl-2-thiazolyl)-2, 5-diphenyl-2H-tetrazolium bromide can be converted to formazan by the mitochondrial reductase of living cells. The 7-mm-diameter T30-4 mycelial disks were cultured on 96-well plates supplemented with DMSO (as control), melatonin (0, 3, 6, 10 mM), Infinito (0, 0.01, 0.05, 0.1 ml/L, ml/L stands for the volume of fungicide in 1 L water), and various combinations of melatonin + Infinito (3 + 0.01, 6 + 0.05, 10 mM + 0.1 ml/L), and incubated at 18°C for 24 h. After 24 h, 10 μL MTT solution was added and incubated at 18°C for 4 h. Then, the samples were measured in an automatic microplate reader working at a wavelength of 490 nm. The cell viability (%) = [A (drug) – A (mock)]/[A (control) – A (mock)].

### Electron microscopy

The T30-4 was cultured in liquid Rye A agar medium containing 6 mM melatonin and the same volumes of DMSO were set as the control, then incubated at 18°C in the dark for 9 days. Next, the mycelia were collected, fixed, observed for scanning electron microscope (SEM) and transmission electron microscope (TEM) as described in the literatures (Cao et al., 2014; Chen et al., 2017).

### Stress tolerance assay

1. The 7-mm-diameter T30-4 mycelial disks were cultured on Rye A agar plates with different supplements: DMSO (as a control), melatonin (3 mM), NaCl (0.1 M)/H_2_O_2_ (0.1 mM), and a combination of melatonin + NaCl/H_2_O_2_. Then the disks were incubated at 18°C in the dark for 14 days.
2. The 7-mm-diameter T30-4 mycelial disks were cultured on Rye A agar plates with different treatments: DMSO (as a control), melatonin (3 mM), UV (1,350 Mw/mm^2^) for 30 mins/37°C for 2 h/4°C for 24 h, and a combination of melatonin + UV/37°C/4°C. Then, the disks were incubated at 18°C in the dark for 14 days.
3. After the 14 days, we measured the diameter of the colony and calculated the inhibition rates. All experiments were repeated three times.

### Analysis of synergism/antagonism effect of the combination of melatonin and fungicide on *P. infestans*

Infinito is a famous fungicide, manufactured by Bayer (Germany), mainly composed of Fluopicolide and Propamocab hydrochloride. The 7-mm-diameter T30-4 mycelial disks were cultured on Rye A agar plates supplemented with DMSO (as control), melatonin solution in DMSO (0, 1, 2, 3 mM), fungicide (0, 0.001, 0.005, 0.01 ml/L, ml/L stands for the volume of fungicide in 1 L water), and various combinations of melatonin + Infinito (1 + 0.001, 1 + 0.005, 1 + 0.01, 2 + 0.001, 2 + 0.005, 2 + 0.01, 3 + 0.001, 3 + 0.005, 3 mM + 0.01 ml/L), and incubated at 18°C for 14 days. Then, the diameters of the colony were measured and the inhibition rates were analyzed by using the Student's *t*-test. Each datum was from the average of three independent biological replicates. The interaction between melatonin and fungicide (Infinito) was quantitatively measured using Combination index (CI) value (Xiong et al., 2017). The levels of reagent interaction were defined following the previous report (Chou, 2006), synergism (CI < 1), additive effect (CI = 1), or antagonism (CI > 1). The percentage of growth value was calculated using the diameter of 14-day-old DMSO-treated control colony (D), and drug-treated colony (T) as follows: [(T-0.7)/(D-0.7)] × 100. IC50 (The half maximal inhibitory concentration) and CI values were calculated by using the CompuSyn software (Chou and Talalay, 1984). The affected value (Fa) indicated the growth inhibition of colony by drug was calculated using the program's instructions as follows: (100 – % growth value)/100.

### Pathogenicity test of *P. infestans* by using the combination of melatonin and fungicide

*P. infestans* strain T30-4 was cultured on Rye A agar at 18°C in the dark for 14 days (Avrova et al., 2003). Then, potato leaves and tuber slices (2 cm × 3 cm × 3 mm in size) were sprayed with DMSO (as control), melatonin solution (3, 6, 8 mM), Infinito (0.0025, 0.025, 0.25 ml/L), and melatonin + Infinito (3 + 0.0025, 3 + 0.025, 3 + 0.25, 6 + 0.0025, 6 + 0.025, 6 + 0.25, 8 + 0.0025, 8 + 0.025, 8 mM + 0.25 ml/L). The 7-mm-diameter T30-4 mycelial disks were put on the potato tuber slices and leaves, and incubated at 18°C with a 12-h/12-h light/dark cycle for 5 days. Then, the size of each lesion was measured and analyzed with the Student's *t*-test. Three biological repeats were performed for each experiment.

### Transcriptome assay and the dataset analysis

The T30-4 was chosen for the RNA-Seq (Haas et al., 2009). The T30-4 was cultured for 14 days in liquid Rye A agar medium at 18°C in the dark. Then the mycelia were transplanted to liquid Rye A agar medium containing with 3 mM melatonin (IC50: half maximal inhibitory concentration) and the same volumes of DMSO as the control, where mycelia were cultured at 18°C in the dark for 24 h. The mycelia were collected and frozen in liquid nitrogen for transcriptome studies. The total RNA was extracted from the samples using the RNAprep Pure Plant Kit (TianGen Biotech, Beijing, China). Three biological replicates were employed in the transcriptome experiments. The sequencing libraries were generated using the NEBNext® Ultra™ RNA Library Prep Kit for Illumina® (NEB, USA) following the manufacturer's recommendations. These libraries were sequenced on an Illumina Hiseq platform and 125 bp/150 bp paired-end reads were generated. Reference genome and gene model annotation files were downloaded from the genome website directly. Paired-end clean reads were aligned to the reference genome using TopHat v2.0.12. HTSeq v0.6.1 was used to count the numbers of reads mapped to each gene. Then, FPKM of each gene was calculated based on the length of the gene and read counts mapped to this gene. Differential expression analysis was performed using the DESeq R package (1.18.0). Genes with an adjusted *p* < 0.05 found by DESeq were regarded as the differentially expressed ones. Gene Ontology (GO) enrichment analysis of differentially expressed genes was implemented by the GO seq R package. GO terms with corrected *p* < 0.05 were considered as significant ones. The KOBAS software was used to test the statistical enrichment of differential expression genes in KEGG pathway. The transcriptome datasets were submitted to NCBI and the accession number—PRJNA415528.

### Real-time quantitative RT-PCR (qRT-PCR) assay

The mycelia were cultured and collected as described in RNA-seq. The total RNA was extracted from the mycelia by using the RNAprep Pure Plant Kit (TianGen Biotech, Beijing, China). The total RNA (1 μg) was used for reverse transcription reaction by using the PrimeScript RT Kit (TAKARA Biotech). After that, the TransStart TopGreen qPCR Super Mix (TransGen Biotech) was used for the qRT-PCR assays by a Bio-Rad CFX96 System. Primer sequences for qRT-PCR were listed in Table S1. Each datum was averaged from three independent biological replicates.

## Results and discussion

### Potato late blight can be significantly attenuated by melatonin *in vivo*

**Figure 1:**
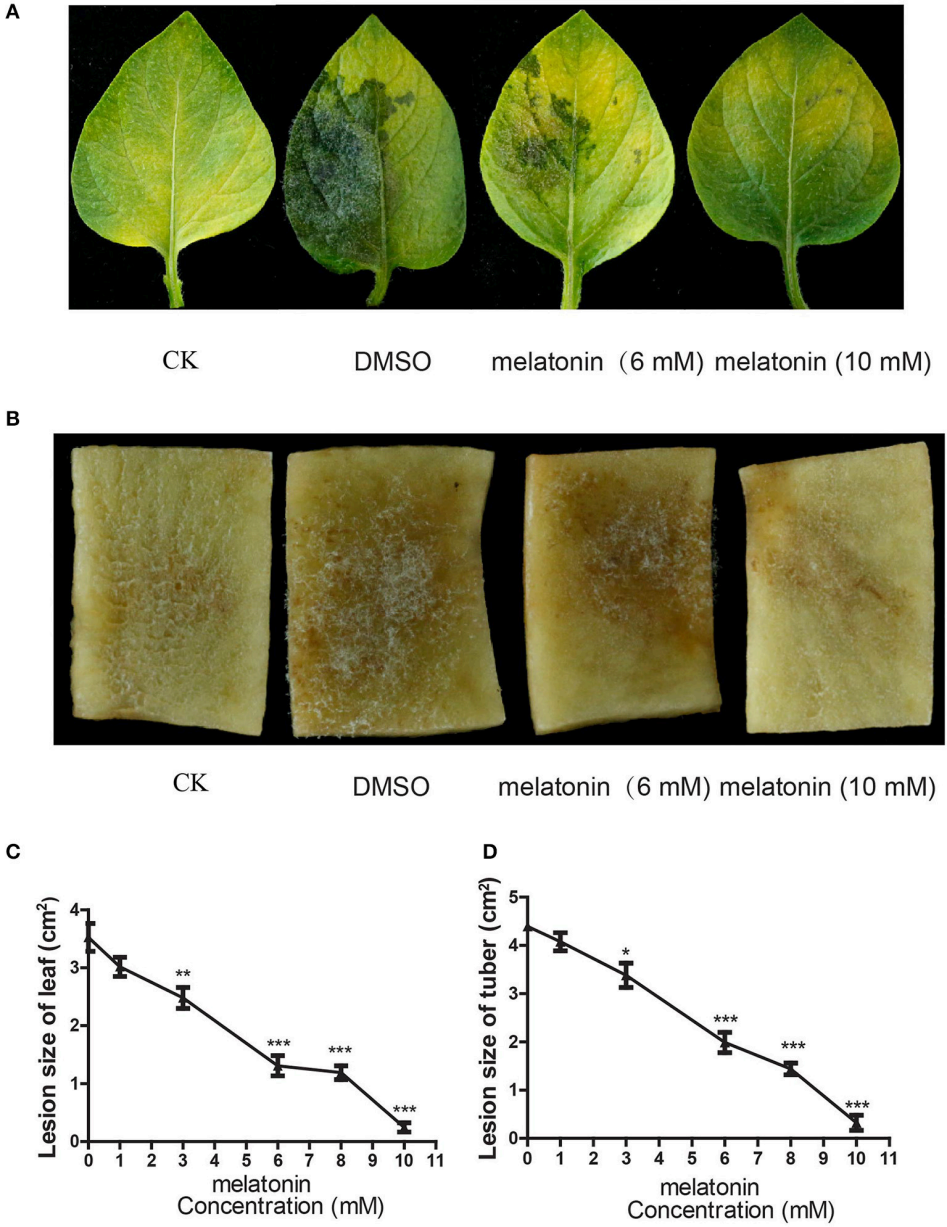
Melatonin suppress potato late blight in a dose dependent manner. **(A,B)** Disease symptoms of potato late blight on leaves **(A)** and fresh tuber slices **(B)** were captured after inoculation of T30-4 strain for 5 days. The leaves and slices were pretreated with water (CK, without inoculation with *P. infestans*), DMSO, and different concentrations of melatonin. **(C,D)** The dose-effect curves of melatonin against *P. infestans* on potato leaves **(C)** and tuber slices **(D)**. (^*^P < 0.05, ^**^P < 0.01, ^***^P < 0.001). Each data point was average of three independent biological replicates.

It has been proven that melatonin displays multiple effects on the cell growth and development of plants, animals and humans (Pandi-Perumal et al., 2006; Reiter et al., 2014), but the impact of melatonin on *P. infestans* has not been explored. For this aim, the T30-4 strain, was inoculated on the potato leaves and tuber slices which were pretreated with DMSO (mock) and various concentrations of melatonin solution. The *in vivo* assay showed that the lesion sizes of leaves and tuber slices infected by *P. infestans* were gradually reduced by increasing melatonin concentrations, and no toxic effects to leaves or tuber even at the highest concentration of melatonin were observed (Figures 1A–D). The occurrence of potato late blight can be prevented on leaves and tuber slices when the concentration of melatonin reached 10 mM (Figures 1A–D). Thus, these *in vivo* observations showed that the potato late blight can be significantly attenuated by using melatonin.

### Suppressing the mycelial growth of *P. infestans* by melatonin *in vitro*

**Figure 2:**
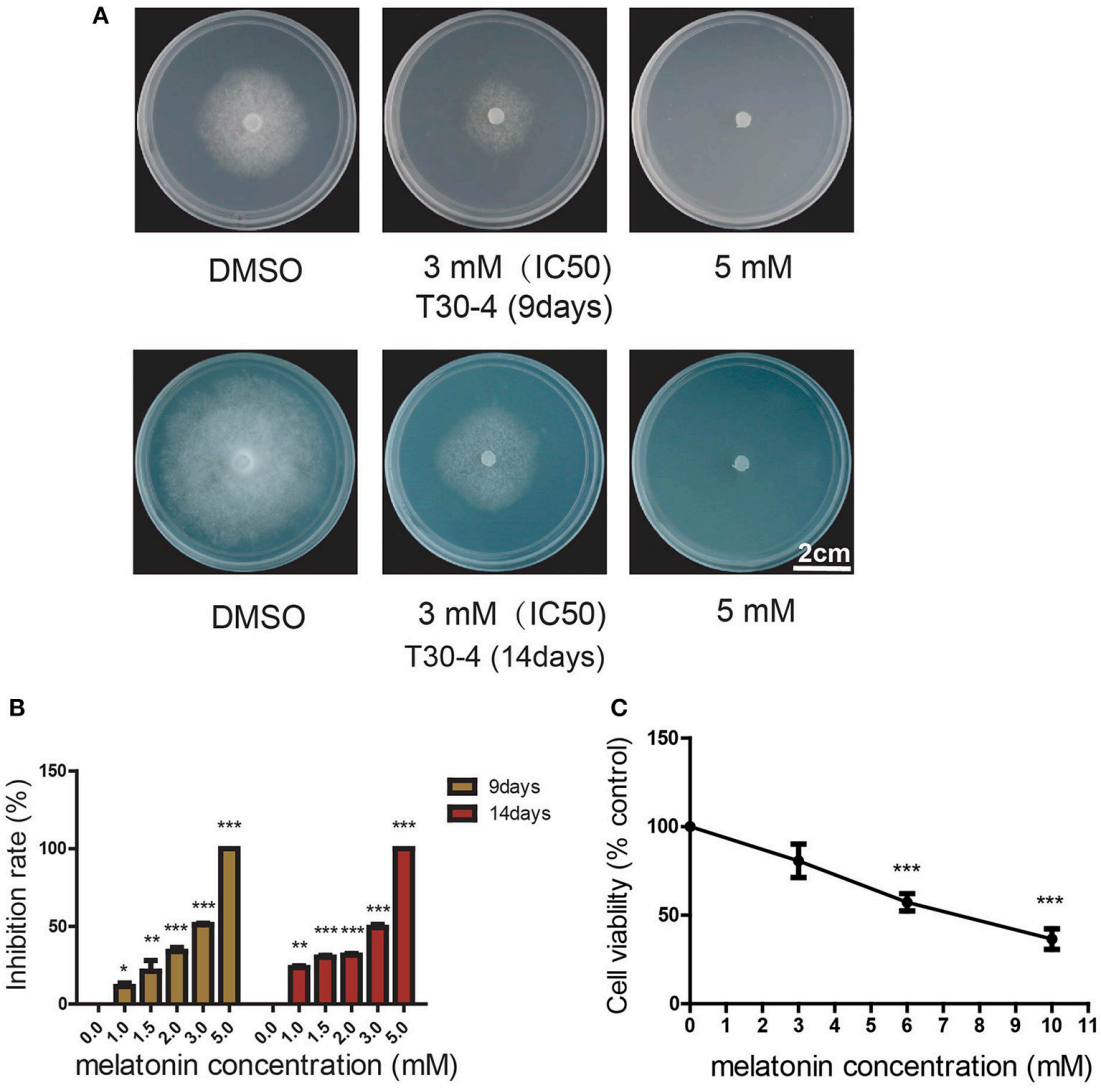
Melatonin can suppress the growth of *P. infestans in vitro*. **(A)** Mycelial phenotypes of *P. infestans* grown on the medium supplemented with melatonin for 9 days (upper panel) and 14 days (below panel). The strain T30-4 was cultured for on Rye A agar amended with 3 or 5 mM melatonin. **(B)** Dose-dependent effect of melatonin on the mycelial inhibition rate of T30-4 at 9 and 14 days. The strain T30-4 was treated with DMSO (control) and melatonin at 1, 1.5, 2, 3, and 5 mM, respectively (^*^P < 0.05, ^**^P < 0.01, ^***^P < 0.001). Each data point was averaged of three independent biological replicates. **(C)** MTT reduction assay showed that cell viability of *P. infestans* T30-4 under melatonin treatment for 24 h. The strain T30-4 was treated with DMSO (control) and melatonin at 3, 6, 10 mM (^*^P < 0.05, ^**^P < 0.01, ^***^P < 0.001).

The previous studies have shown that melatonin can act as an important signal to induce innate immunity in plants (Yin et al., 2013; Lee et al., 2014, 2015; Qian et al., 2015; Shi et al., 2015, 2016; Zhao et al., 2015; Lee and Back, 2016; Wei et al., 2017). To exclude the effects of plant immunity induced by melatonin on the occurrence of potato late blight, we next investigated whether melatonin can directly suppress the cell growth of *P. infestans*. Toward this objective, the strain T30-4 was cultured on the medium supplemented with different concentrations of melatonin. When higher concentrations of melatonin were used, slower growth of mycelia was observed (Figures 2A,B). The IC50 of melatonin appeared at 3 mM for T30-4 (Figures 2A,B). The mycelial growth was completely inhibited at a melatonin concentration of 5 mM (Figures 2A,B). Furthermore, after being incubated for 24 h with different concentrations of melatonin, the mycelial cell viability was tested by the MTT reduction assay. This assay showed that the cell viability was decreased with increases in melatonin concentration (Figure 2C). These *in vitro* observations suggested that melatonin could directly suppress the cell growth of *P. infestans* to attenuate the potato late blight, which was consistent with the previous studies that exogenous melatonin directly resists the human pathogens *in vitro*, including *Leishmania infantum, Candida parapsilosis, Staphylococcus aureus* (Tekbas et al., 2008; Elmahallawy et al., 2014; Yang et al., 2014).

### Influencing the ultrastructure of *P. infestans* by melatonin

**Figure 3:**
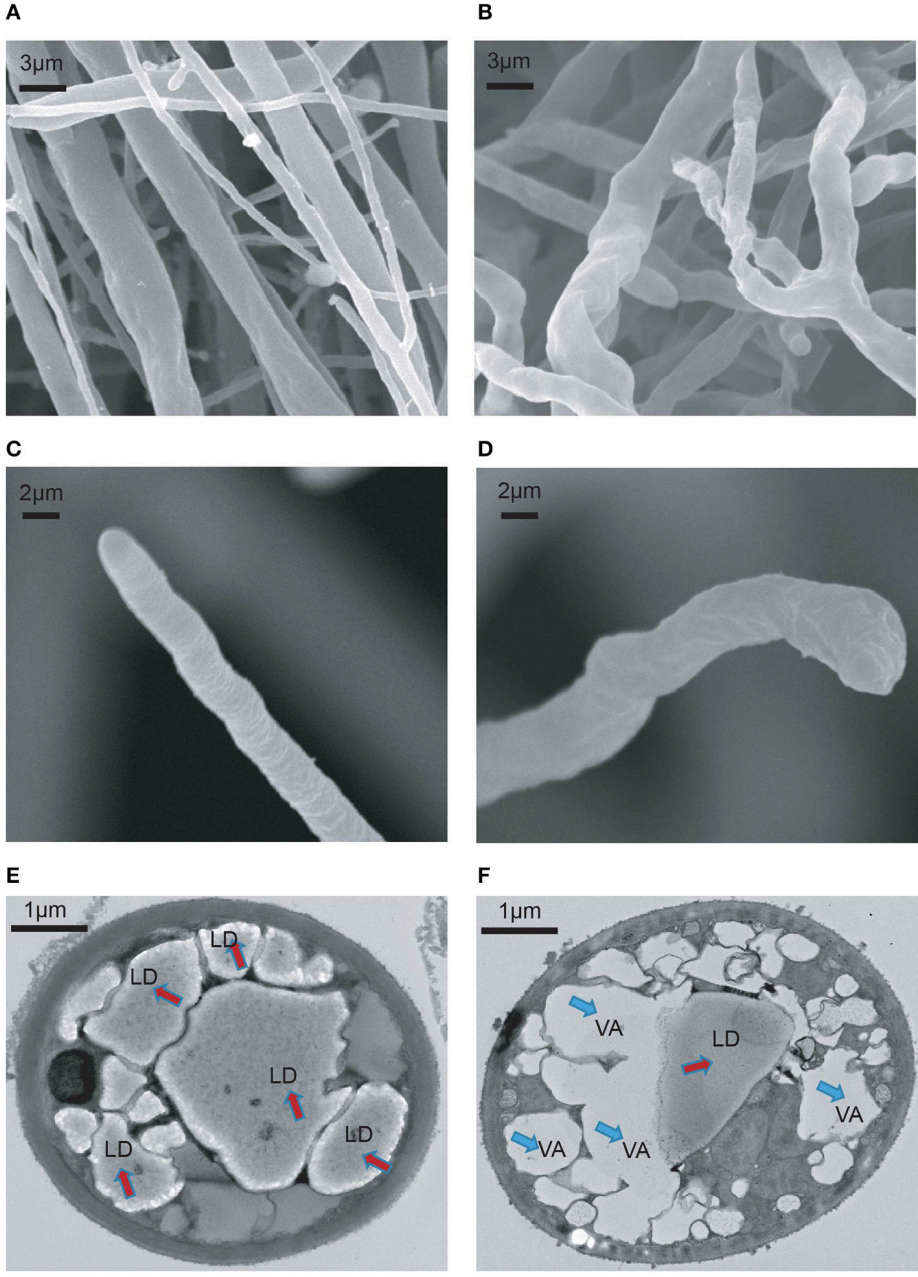
SEM and TEM analysis of melatonin treated *P. infestans*. Ultrastructure of **(A)** hyphal surface untreated by melatonin documented by SEM, **(B)** hyphal surface treated by melatonin documented by SEM, **(C)** hyphal tip untreated by melatonin documented by SEM, **(D)** hyphal tip treated by melatonin documented by SEM, **(E)** hypha untreated by melatonin documented by TEM. LP, lipid droplet, **(F)** hypha treated by melatonin documented by TEM. VA, vacancy.

To understand the growth inhibition phenotypes of *P. infestans* caused by melatonin treatment, the cell ultrastructure of the melatonin-treated *P. infestans* was investigated using scanning electron microscopy (SEM) and transmission electron microscopy (TEM). As shown in Figures 3A,B, the disorganized hyphal growth pattern, and aberrant and distorted hyphae were observed in melatonin-treated *P. infestans* in comparison with the untreated controls; this could result in the inhibition of hyphal growth of *P. infestans*. In melatonin-treated *P. infestans*, the branched hyphae and blunt hyphal tip (Figures 3B,D), possibly was a result of suppressing the apical dominance of the hyphal tip that could lead to the inhibition of hyphal elongation (Semighini and Harris, 2008; Zhao et al., 2011). Furthermore, the TEM analysis showed that more vacancies and fewer lipid droplets appeared in the cells of *P. infestans* with melatonin treatment compared to the control (Figures 3E,F), indicating that the melatonin treatment resulted in the decrease of lipid droplets in *P. infestans*. It is well-known that lipid droplets play an important role in various biological processes, such as facilitation of cell growth, propagation as well as infection of fungus, viral and bacterium (Bago et al., 2002; Stehr et al., 2012; Radulovic et al., 2013; Kobae et al., 2014; Bi et al., 2016). Thus, the reduction of lipid droplets could result in the inhibition of growth of *P. infestans*.

### Enhanced susceptibility of *P. infestans* to various environmental stresses in the presence of melatonin

**Figure 4:**
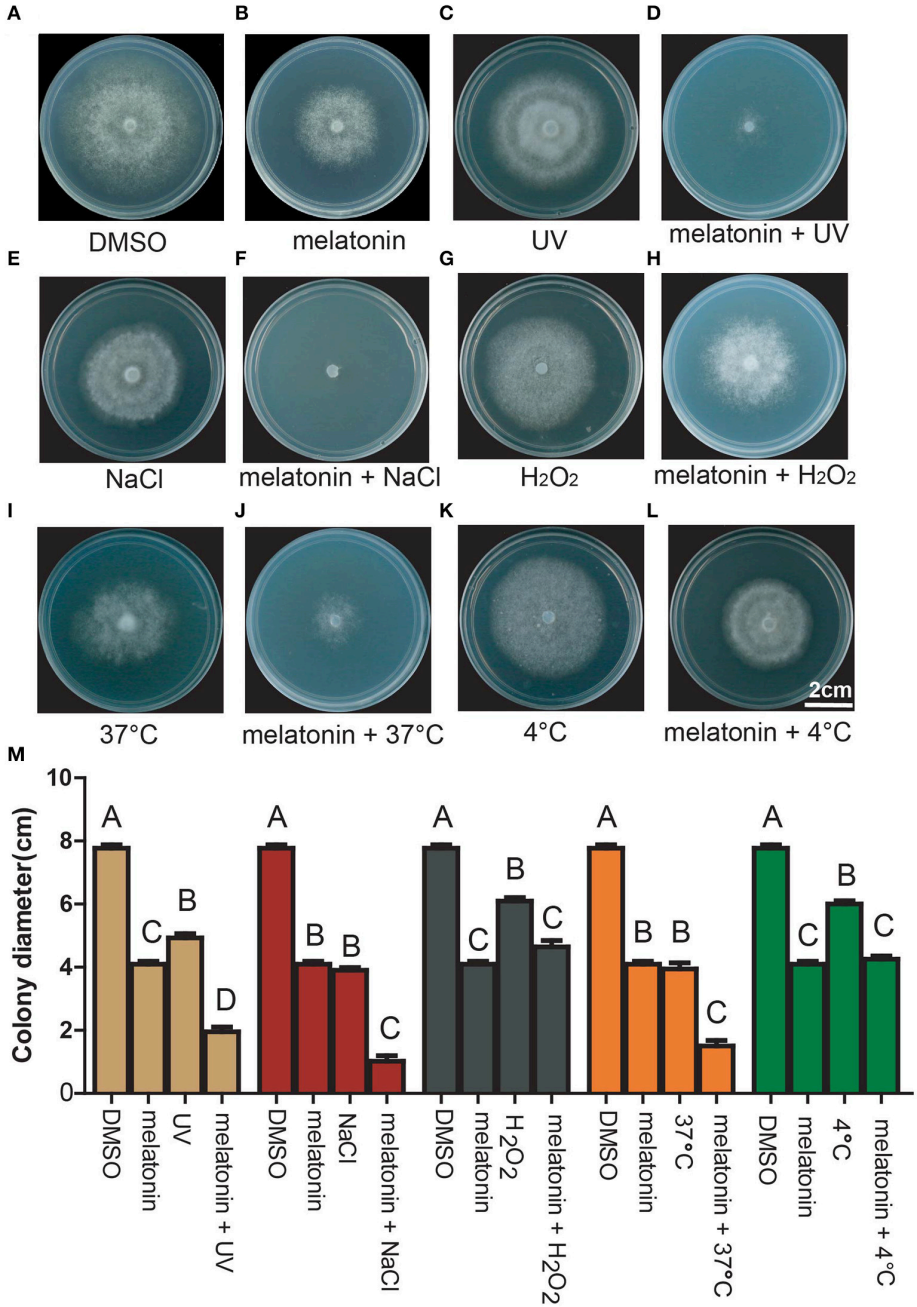
Growth pattern of *P. infestans* in response to the treatment of DMSO, melatonin, stress, and melatonin + stress. Colony morphology of *P. infestans* grown on Rye A agar medium treated by **(A–L)** DMSO, 3 mM melatonin, UV, 3 mM melatonin + UV, 0.1 M NaCl, 3 mM melatonin + 0.1 M NaCl, 0.1 mM H_2_O_2_, 3 mM melatonin + 0.1 mM H_2_O_2_, 37°C, 3 mM melatonin + 37°C, 4°C, 3 mM melatonin + 4°C. **(M)** Colony diameter of *P. infestans* grown on Rye A agar medium treated by DMSO, 3 mM melatonin, UV/NaCl/H_2_O_2_/37°C/4°C, 3 mM melatonin + UV/NaCl/H_2_O_2_/37°C/4°C. Capitalized letters indicate significant difference, *p* < 0.01.

The tolerance of *P. infestans* to environmental stresses derived from salt, heat, cold and Ultra Violate (UV) radiation can promote the successful occurrence and epidemics of potato late blight (Ortizurquiza and Keyhani, 2014). We therefore explored the tolerance of *P. infestans* to these stresses and the combination of these stresses with melatonin. The mycelial growth of *P. infestans* was inhibited when using salt, UV, heat, H_2_O_2_ and cold, respectively (Figures 4A–C,E,G,I,K,M), indicating that *P. infestans* was sensitive to these types of environmental stresses. Interestingly, the mycelial growth of *P. infestans* was more severely inhibited by the pairwise combination of melatonin and these stresses (UV/salt/heat) than that of melatonin or any of these stresses alone (Figures 4A–F,I,J,M). These results indicated that melatonin reduced the stress tolerance of *P. infestans* under challenging environmental conditions.

### Melatonin and chemical fungicide synergistically inhibit the growth of *P. infestans in vitro*

**Figure 5:**
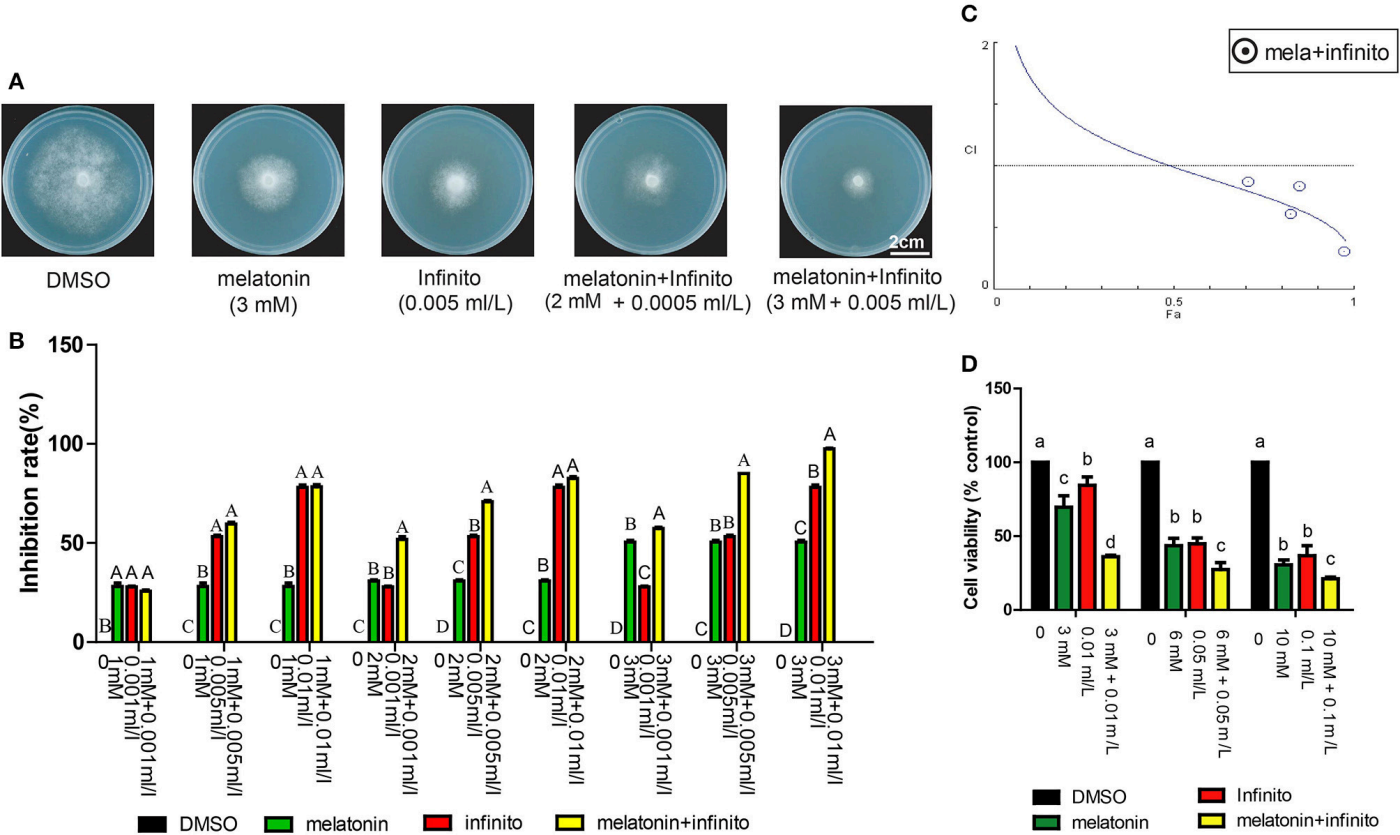
Synergistic growth inhibition of *P. infestans* induced by co-application of the melatonin and Infinito. **(A)** Colony morphology on Rye A agar medium that contains DMSO, 3 mM melatonin (IC50), 0.005 ml/L Infinito (IC50), 2 mM melatonin + 0.0005 ml/L Infinito (IC50), 3 mM melatonin + 0.005 ml/L Infinito. **(B)** Mycelial inhibition rate of *P. infestans* T30-4 under DMSO, melatonin, Infinito, and melatonin + Infinito treatment for 14 days. Each data point was averaged from three independent biological replicates. Capitalized letters indicate significant difference, *p* < 0.01. **(C)** Fa-CI curve showed synergism (CI < 1) generated by melatonin + Infinito treatment. **(D)** MTT reduction assay showed that cell viability of *P. infestans* T30-4 under DMSO, melatonin, Infinito, and melatonin + Infinito treatment for 24 h. Lower case letters indicate significant difference, *p* < 0.05.

Currently, the control of potato late blight mainly depends on high dosage and frequency of fungicide applications. These practices result in many health and environmental issues. Reduced fungicide doses are important for a sustainable environment. It has been reported that melatonin could be used as a synergist to reduce the use of some drugs and enhance the drug effect, for example, vincristine, atorvastatin, vitamin C and E (Lissoni et al., 1999; Gitto et al., 2001; Casadozapico et al., 2010; Dayoub et al., 2011). We therefore examined whether the synergistic effects exist in combined application of melatonin and fungicide against *P. infestans*. Infinito is one of the most effective, expensive and extensively used chemical fungicides for potato late blight control. The results showed that the inhibitory effects of T30-4 treated with Infinito + melatonin was more significantly enhanced than that of Infinito or melatonin alone (Figures 5A,B); this suggested that melatonin can promote *P. infestans* to become more susceptible to Infinito. The IC50s of melatonin, Infinito, and melatonin + Infinito were also examined, and the values were 3 mM, 0.005 ml/L, 2 mM + 0.0005 ml/L, respectively. A significant reduction of Infinito dose (10-fold reduction) was generated by co-applying melatonin (Figure 5A), which indicated that the chemical fungicide dose was decreased when combined with melatonin for potato late blight control. Moreover, CI < 1 was observed, confirming that the synergistic effects exist in the melatonin + Infinito (Figure 5C). The MTT reduction assay also confirmed that the cell viability of T30-4 treated with Infinito + melatonin was more significantly decreased than that of Infinito or melatonin alone (Figure 5D). Thus, these observations provide new insights into the synergistic anti-fungal effects of Infinito with melatonin on *P. infestans*, and expand the understanding of the synergistic effect of melatonin (Lissoni et al., 1999; Casadozapico et al., 2010; Dayoub et al., 2011).

### Melatonin reduced the fungicide dose for potato late blight control *in vivo*

**Figure 6:**
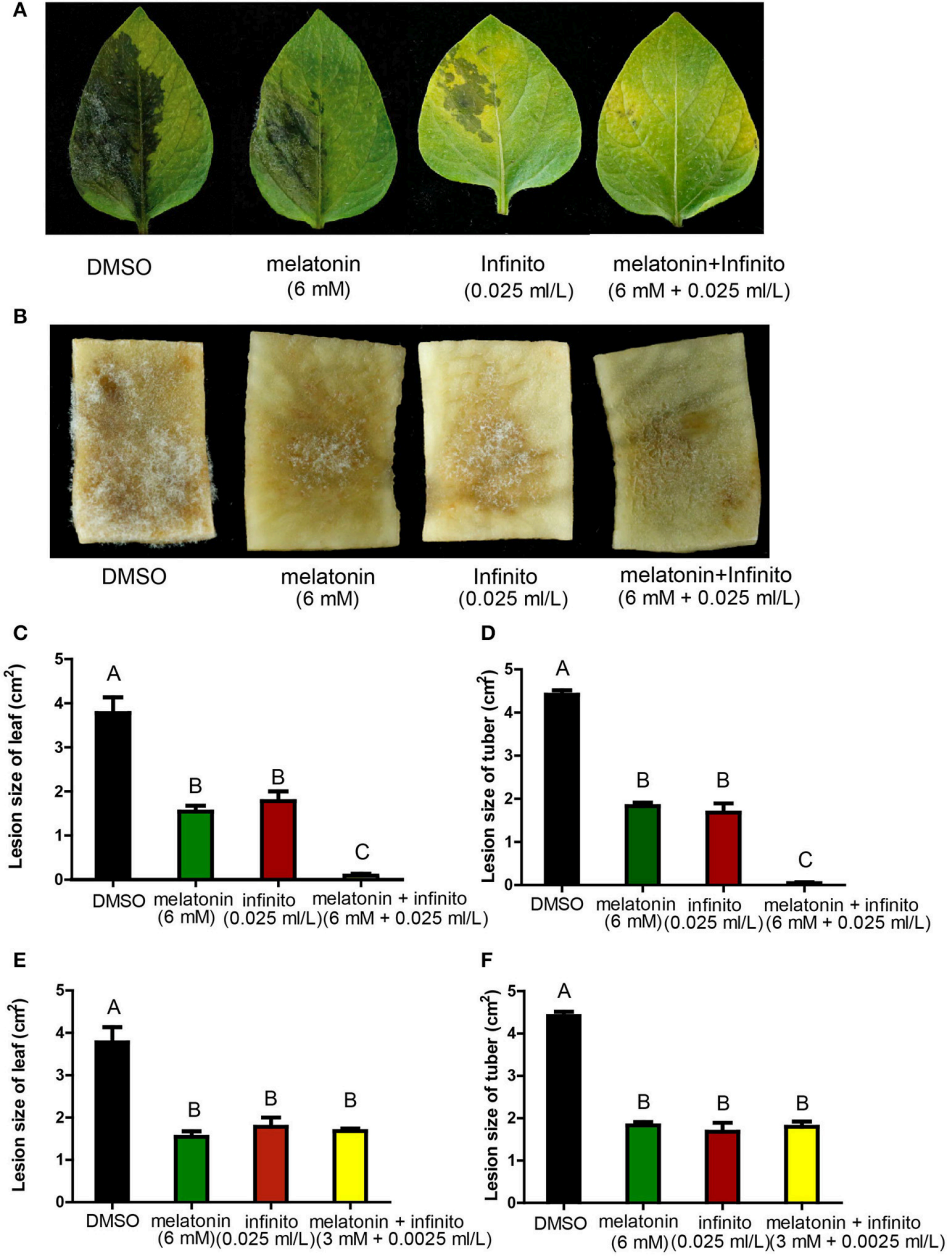
Disease symptoms of potato late blight on leaves and tubers slices infected by *P. infestans* for 5 days in the presence of melatonin and Infinito. **(A)** Disease symptoms of potato leaves with the treatment of DMSO, 6 mM melatonin, 0.025 ml/L Infinito, 6 mM melatonin + 0.025 ml/L Infinito, respectively, after 5 days. **(B)** Disease symptoms of potato tuber slices with the treatment of DMSO, 6 mM melatonin, 0.025 ml/L Infinito, 6 mM melatonin + 0.025 ml/L Infinito, respectively, after 5 days. **(C)** Lesion size of leaves with the treatment of 6 mM melatonin, 0.025 ml/L Infinito, 6 mM melatonin + 0.025 ml/L Infinito, respectively, after 5 days. **(D)** Lesion size of tuber slices with the treatment of 6 mM melatonin, 0.025 ml/L Infinito, 6 mM melatonin + 0.025 ml/L Infinito, respectively, after 5 days. **(E)** The lesion size of leaves infected by *P. infestans* treated with 6 mM melatonin, 0.025 ml/L Infinito, 3 mM melatonin + 0.0025 ml/L Infinito, respectively, after 5 days. **(F)** The lesion size of tuber slices under the treatment of 6 mM melatonin, 0.025 ml/L Infinito, 3 mM melatonin + 0.0025 ml/L Infinito, respectively, after 5 days. Each data point was averaged of three independent biological replicates. Capitalized letters indicate significant difference, *p* < 0.01

Next, the *in vivo* assay of synergistic inhibition was conducted. When treated with melatonin + Infinito, the lesion sizes infected by *P. infestans* on the potato leaves and tuber slices were much smaller than that of melatonin or Infinito alone (Figures 6A–D, and Figures S1A,B). The 6 mM melatonin + 0.025 ml/L Infinito was the optimal concentration among these combinations (the lowest concentration of Infinito for reaching the same small lesion size, while the concentration of melatonin was modest) (Figures S1A,B). A significant reduction of Infinito dose (10-fold reduction) was achieved by the combination with melatonin *in vivo* (Figures 6E,F). This result revealed an eco-friendly potential for the reduced use of fungicides by co-application of melatonin in potato late blight control.

### Analysis of melatonin effects on *P. infestans* by the transcriptome

**Figure 7:**
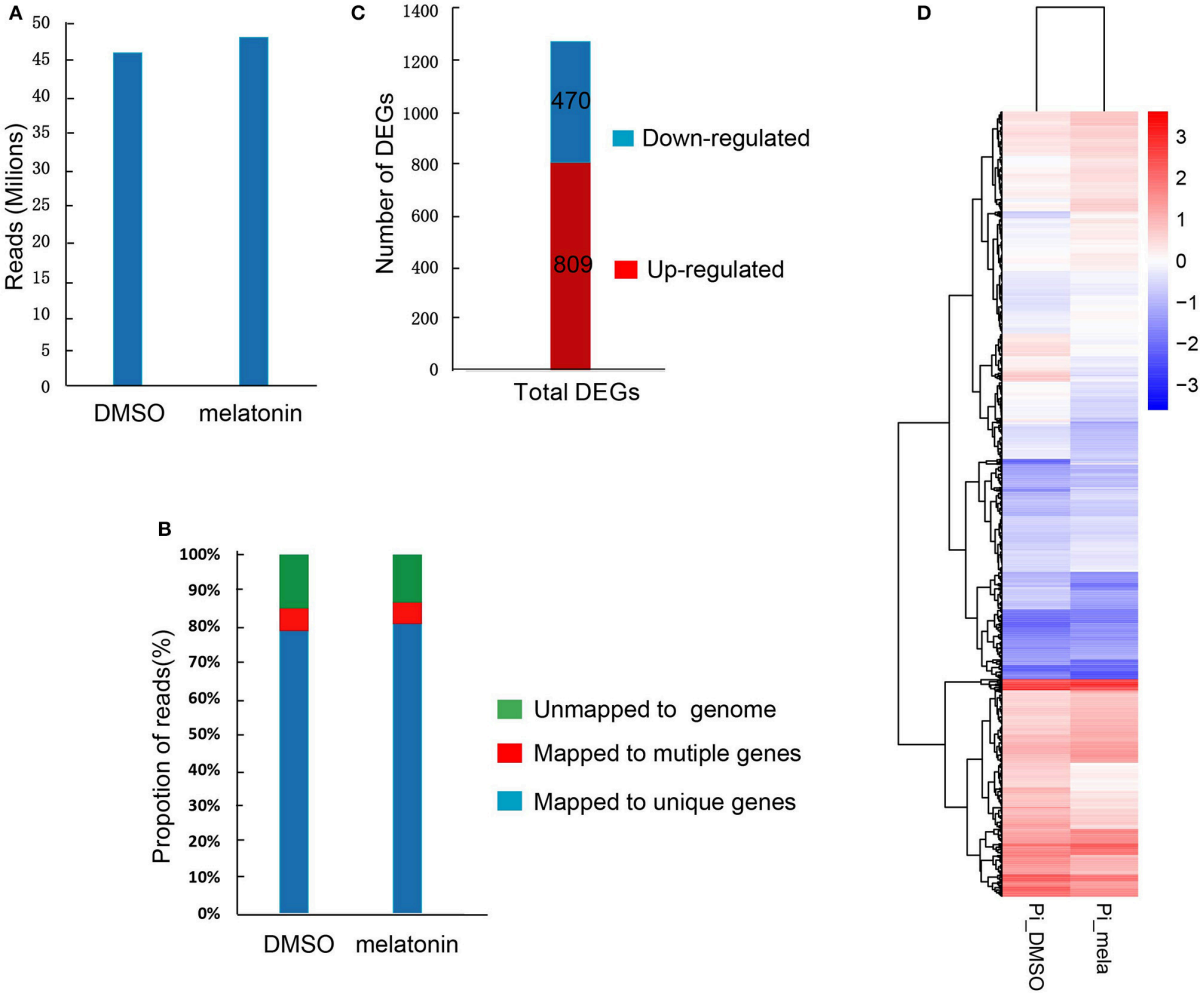
Summary of the basic information for the transcriptome data. **(A)** High-quality clean reads from high-throughput sequencing. **(B)** Proportions of high-quality clean reads of unmapped to genes, mapped to unique genes and multiple genes. **(C)** The number of differentially expressed genes (DEGs). **(D)** The heat-map of DEGs induced by melatonin.

To further investigate the underlying molecular mechanism about the roles of melatonin in the growth and development of *P. infestans*, we performed transcriptome analysis to assess the global transcriptional profile revealing differentially expressed genes (DEGs) induced by melatonin. The T30-4 strain was treated with DMSO (mock) and 3 mM melatonin for 24 h and the samples were collected for transcriptome assay. After trimming for quality and adapter sequences, 45.79 and 47.89 million transcriptome reads were obtained with the treatments of 3 mM melatonin and DMSO as the control, respectively (Figure 7A), of which 85.73, 5.88, and 79.85% reads could be mapped to the annotated genome, multiple genes, and unique genes of *P. infestans*, respectively (Figure 7B). 1,279 DEGs out of 10,844 detected genes were identified in the melatonin- treated samples in contrast to DMSO-treated one (Table S2). Among these DEGs, 809 were significantly up-regulated and 470 significantly down-regulated (Figure 7C). The heat-map also showed that melatonin treatment was tend to increase the gene expression level (FPKM) of the DEGs (Figure 7D and Figure S2, Table S3).

To investigate the detailed functions of melatonin's effects on *P. infestans*, the GO terms and KEGG pathways were analyzed. In the transcriptome, among the top 30 GO terms and KEGG pathways (sorted by their *p*-values in the descending order; Tables S4A,B, S5A,B), there were 19 GO terms and 26 KEGG pathways associated with the metabolism of carbohydrates, lipids, nucleic acid, amino acid, cofactors and vitamins (Tables 1, 2). Notably, among 19 GO terms and 26 KEGG pathways, the down-regulated genes were predominated in 14 GO terms and 14 KEGG pathways (Table 3), suggesting that the various metabolic processes in *P. infestans* are dramatically suppressed after melatonin treatment.

**Table 1:** Nineteen GO terms related to metabolism in top30 GO terms in transcriptome.

**Description**	**Classification**	**Number of genes up regulated**	**Number of genes down regulated**
Organic acid metabolic process	Organic acid metabolic process	27	30
Oxoacid metabolic process	Organic acid metabolic process	27	30
Carboxylic acid metabolic process	Organic acid metabolic process	27	29
Pyruvate metabolic process	Organic acid metabolic process	0	12
Branched-chain amino acid biosynthetic process	Cellular amino acid metabolic process	0	8
Cellular amino acid biosynthetic process	Cellular amino acid metabolic process	17	8
Branched-chain amino acid metabolic process	Cellular amino acid metabolic process	0	8
ATP generation from ADP	Nucleotide metabolic process	0	11
ADP metabolic process	Nucleotide metabolic process	0	11
Purine ribonucleoside diphosphate metabolic process	Nucleoside phosphate metabolic process	0	11
Ribonucleoside diphosphate metabolic process	Nucleoside phosphate metabolic process	0	11
Purine nucleoside diphosphate metabolic process	Nucleoside phosphate metabolic process	0	11
rRNA processing	Nucleic acid metabolic process	14	0
rRNA metabolic process	Nucleic acid metabolic process	14	0
ncRNA processing	Nucleic acid metabolic process	22	0
RNA processing	Nucleic acid metabolic process	32	0
Oxidation-reduction process	Single-organism metabolic process	44	45
Single-organism carbohydrate catabolic process	Carbohydrate catabolic process	1	11
Glycolytic process	Carbohydrate catabolic process	0	11

*The classification based on quick GO, website: http://www.ebi.ac.uk/QuickGO/*.

**Table 2:** Twenty-six KEGG pathways related to metabolism in top30 KEGG pathways in transcriptome.

**Description**	**Classification**	**Number of genes up regulated**	**Number of genes down regulated**
Glycolysis/Gluconeogenesis	Metabolism; Carbohydrate metabolism	4	16
Pyruvate metabolism	Metabolism; Carbohydrate metabolism	4	6
Galactose metabolism	Metabolism; Carbohydrate metabolism	0	4
Fructose and mannose metabolism	Metabolism; Carbohydrate metabolism	0	3
Pentose and glucuronate interconversions	Metabolism; Carbohydrate metabolism	5	1
Ascorbate and aldarate metabolism	Metabolism; Carbohydrate metabolism	3	0
Glycerolipid metabolism	Metabolism; Lipid metabolism	4	6
Alanine, aspartate and glutamate metabolism	Metabolism; Amino acid metabolism	6	1
Cysteine and methionine metabolism	Metabolism; Amino acid metabolism	4	0
Valine, leucine and isoleucine degradation	Metabolism; Amino acid metabolism	6	0
Valine, leucine and isoleucine biosynthesis	Metabolism; Amino acid metabolism	0	4
Arginine and proline metabolism	Metabolism; Amino acid metabolism	9	1
Glutathione metabolism	Metabolism; Metabolism of other amino acids	3	4
Cyanoamino acid metabolism	Metabolism; Metabolism of other amino acids	3	3
Beta-Alanine metabolism	Metabolism; Metabolism of other amino acids	3	0
Taurine and hypotaurine metabolism	Metabolism; Metabolism of other amino acids	1	1
Pyrimidine metabolism	Metabolism; Nucleotide metabolism	19	2
Purine metabolism	Metabolism; Nucleotide metabolism	19	5
Biosynthesis of amino acids	Metabolism; Overview	9	16
Carbon metabolism	Metabolism; Overview	4	20
Degradation of aromatic compounds	Metabolism; Overview	1	2
2-Oxocarboxylic acid metabolism	Metabolism; Overview	2	5
Pantothenate and CoA biosynthesis	Metabolism; Metabolism of cofactors and vitamins	0	5
Porphyrin and chlorophyll metabolism	Metabolism; Metabolism of cofactors and vitamins	2	3
Folate biosynthesis	Metabolism; Metabolism of cofactors and vitamins	3	0
Nitrogen metabolism	Metabolism; Energy metabolism	2	3

*The classification based on the KEGG pathway database, http://www.genome.jp/kegg/*.

**Table 3:** The prominent down-regulated genes in 14 GO terms and 14 KEGG pathways related to metabolism in transcriptome.

	**Classification**	**Number of genes up regulated**	**Number of genes down regulated**
**GO TERM**			
Organic acid metabolic process	Organic acid metabolic process	27	30
Oxoacid metabolic process	Organic acid metabolic process	27	30
Carboxylic acid metabolic process	Organic acid metabolic process	27	29
Pyruvate metabolic process	Organic acid metabolic process	0	12
Branched-chain amino acid biosynthetic process	Cellular amino acid metabolic process	0	8
Branched-chain amino acid metabolic process	Cellular amino acid metabolic process	0	8
ATP generation from ADP	Nucleotide metabolism	0	11
ADP metabolic process	Nucleotide metabolism	0	11
Purine ribonucleoside diphosphate metabolic process	Nucleotide metabolism	0	11
Ribonucleoside diphosphate metabolic process	Nucleotide metabolism	0	11
Purine nucleoside diphosphate metabolic process	Nucleotide metabolism	0	11
Oxidation-reduction process	Single-organism metabolic process	44	45
Single-organism carbohydrate catabolic process	Carbohydrate catabolic process	1	11
Glycolytic process	Carbohydrate catabolic process	0	11
**KEGG PATHWAY**			
Glycolysis/Gluconeogenesis	Metabolism; Carbohydrate metabolism	4	16
Pyruvate metabolism	Metabolism; Carbohydrate metabolism	4	6
Galactose metabolism	Metabolism; Carbohydrate metabolism	0	4
Fructose and mannose metabolism	Metabolism; Carbohydrate metabolism	0	3
Glycerolipid metabolism	Metabolism; Lipid metabolism	4	6
Valine, leucine and isoleucine biosynthesis	Metabolism; Amino acid metabolism	0	4
Glutathione metabolism	Metabolism; Metabolism of other amino acids	3	4
Biosynthesis of amino acids	Metabolism; Overview	9	16
Carbon metabolism	Metabolism; Overview	4	20
Degradation of aromatic compounds	Metabolism; Overview	1	2
2-Oxocarboxylic acid metabolism	Metabolism; Overview	2	5
Pantothenate and CoA biosynthesis	Metabolism; Metabolism of cofactors and vitamins	0	5
Porphyrin and chlorophyll metabolism	Metabolism; Metabolism of cofactors and vitamins	2	3
Nitrogen metabolism	Metabolism; Energy metabolism	2	3

### Differentially expressed genes related to amino acid metabolism of *P. infestans* were significantly affected by melatonin

Among these altered metabolic processes in transcriptome (top 30 GO terms and KEGG pathways), the number of GO terms and KEGG pathways related to amino acid metabolism was the largest (three GO terms and ten KEGG pathways) (Table 4, Table S6A). This result indicated that the amino acid metabolism was the most significantly targeted metabolic process by melatonin. Among these GO terms and KEGG pathways associated with amino acid metabolism (Tables S6A,B), the biosynthesis of amino acids was the overview pathway in which the down-regulated genes were predominant (Table 3). Particularly, the genes encoding rate-limiting enzymes: pyruvate kinase, fructose-bisphosphate aldolase, isocitrate dehydrogenase [NADP] were down-regulated as well (Table 5, Tables S6A,B). The genetic mutation of these rate-limiting enzymes can reduce amino acid metabolism levels (Heneka et al., 2001; Malay et al., 2005; Gupta and Bamezai, 2010; Grace et al., 2015; Lv et al., 2017), indicating that the amino acid metabolism was suppressed after melatonin treatment. Collectively, the results and observations from the transcriptome suggest that amino acid metabolism of *P. infestans* is likely the main target of melatonin.

**Table 4:** The GO term or KEGG pathway related to metabolism of amino acid, carbohydrate, nucleic acid, lipid.

**Amino acid metabolic process**	**GO Term**	
	**Amount**	**Description**
	3	Branched-chain amino acid biosynthetic process
		Cellular amino acid biosynthetic process
		Branched-chain amino acid metabolic process
	**KEGG pathway**	
	**Amount**	**Description**
	10	Alanine, aspartate and glutamate metabolism
		Cysteine and methionine metabolism
		Valine, leucine and isoleucine degradation
		Valine, leucine and isoleucine biosynthesis
		Arginine and proline metabolism
		Glutathione metabolism
		Cyanoamino acid metabolism
		Beta-Alanine metabolism
		Taurine and hypotaurine metabolism
		Biosynthesis of amino acids
**Nucleic acid metabolic process**	**GO Term**	
	**Amount**	**Description**
	4	rRNA processing
		rRNA metabolic process
		ncRNA processing
		RNA processing
**Nucleotide metabolic process**	**GO Term**	
	**Amount**	**Description**
	2	ATP generation from ADP
		ADP metabolic process
	**KEGG pathway**	
	**Amount**	**Description**
	2	Pyrimidine metabolism
		Purine metabolism
**Nucleoside phosphate metabolic process**	**GO Term**	
	**Amount**	**Description**
	3	Ribonucleoside diphosphate metabolic process
		Purine nucleoside diphosphate metabolic
		Purine ribonucleoside diphosphate metabolic process
**Organic acid metabolic process**	**GO Term**	
	**Amount**	**Description**
	4	Organic acid metabolic process
		Oxoacid metabolic process
		Carboxylic acid metabolic process
		Pyruvate metabolic process
**Carbohydrate metabolic process**	**GO Term**	
	**Amount**	**Description**
	2	Glycolytic process
		Single-organism carbohydrate catabolic process
	**KEGG pathway**	
	**Amount**	**Description**
	6	Glycolysis/Gluconeogenesis
		Pyruvate metabolism
		Galactose metabolism
		Fructose and mannose metabolism
		Pentose and glucuronate interconversions
		Ascorbate and aldarate metabolism
**Lipid metabolism**	**KEGG pathway**	
	**Amount**	**Description**
	1	Glycerolipid metabolism
**Cofactors and vitamins metabolism**	**KEGG pathway**	
	**Amount**	**Description**
	3	Pantothenate and CoA biosynthesis
		Porphyrin and chlorophyll metabolism
		Folate biosynthesis

**Table 5:** The DEG related to biosynthesis of amino acid.

**Gene ID**	**log2FC**	**Padj**	**Interpro description**
**BIOSYNTHESIS OF AMINO ACIDS**			
PITG_13402	0.80214	2.88E-06	Cystathionine beta-lyase
Novel01245	−0.76229	0.015307	
PITG_01940	−1.0332	1.46E-06	Triosephosphate isomerase/glyceraldehyde-3-phosphate dehydrogenase, putative
PITG_17516	0.70944	0.000041	S-adenosylmethionine synthase
PITG_14696	0.4306	0.036763	Serine hydroxymethyltransferase
PITG_20970	−0.52586	0.023256	Triosephosphate isomerase/glyceraldehyde-3-phosphate dehydrogenase, putative
PITG_03598	−0.96851	5.02E-09	6-phosphofructokinase, putative
PITG_03599	0.61407	0.000949	Alanine aminotransferase 2
PITG_13116	−1.3446	2.08E-17	Triosephosphate isomerase
PITG_02049	0.56454	0.003107	Citrate synthase
PITG_12161	0.51372	0.011304	Delta-1-pyrroline-5-carboxylate synthetase
PITG_23158	−0.4919	0.018664	Isocitrate dehydrogenase [NADP]
PITG_01938	−1.0026	0.002778	Glyceraldehyde-3-phosphate dehydrogenase
PITG_01939	−0.93518	0.023748	Glyceraldehyde-3-phosphate dehydrogenase, putative
PITG_07400	−0.92768	2.63E-08	Phosphoglycerate mutase
PITG_21697	−0.95046	1.09E-07	conserved hypothetical protein
PITG_22685	−1.1672	2.27E-12	Dihydroxy-acid dehydratase, putative
PITG_02925	−1.4579	1.13E-20	Ketol-acid reductoisomerase
PITG_02786	−0.90929	6.57E-07	Fructose-bisphosphate aldolase
PITG_02740	−0.5491	0.004195	Acetolactate synthase
PITG_09393	−0.55962	0.002885	Pyruvate kinase
PITG_20759	−0.50352	0.019167	Dihydroxy-acid dehydratase
PITG_00221	0.53793	0.010979	Tryptophan synthase, putative
PITG_02210	1.2957	6.82E-14	Phenylalanine-4-hydroxylase, henna-like protein
PITG_05374	0.62953	0.000536	Argininosuccinate synthase

Carbohydrate metabolism and lipid metabolism were also altered in transcriptome (Tables 1, 2, 4 and Tables S6C,D). For example, the down-regulation of the rate-limiting enzyme 6-phosphofructokinase (PFK) in the transcriptome (Table S6C) would reduce the carbohydrate metabolism levels of *P. infestans* by melatonin (Raben et al., 1995; Wegener and Krause, 2002). The down-regulation of another rate-limiting enzyme fatty acid synthase (FASN) in transcriptome (Table S6D) documented that the suppression of lipid metabolism of *P. infestans* by melatonin (Smith et al., 2003), which is possibly the reason for the decrease in lipid droplets observed in TEM analysis of melatonin-treated samples.

In summary, the transcriptome analysis showed that multiple metabolic processes were affected by melatonin in *P. infestans*, especially amino acid metabolism. The perturbation of amino acid metabolism leads to cell death (Mizushima, 2005; Green et al., 2014), indicating that the disruption of amino acid metabolism homeostasis by melatonin may significantly contribute to the inhibition of growth in *P. infestans*. The previous study also showed that amino acid metabolism of *P. infestans* may be an important metabolic target for fungicides (Grenvillebriggs et al., 2005). Additionally, the amino acid metabolism has been a target to control the plant pathgens by some fungicides, such as pyrimethanil (Milling and Richardson, 1995). Thus, the results from this study imply that the target of amino acid metabolism in *P. infestans* by melatonin could be a novel alternative for the biocontrol of potato late blight.

### Differentially expressed genes related to stress tolerance of *P. infestans* were affected by melatonin

Though metabolic processes in *P. infestans* were significantly suppressed after melatonin treatment, the expression of some DEGs related to stress tolerance was also affected by melatonin. The transcriptome data showed that 15 DEGs were related to stress tolerance (Table 6). Among them, 9 DEGs were down-regulated and 6 DEGs were up-regulated. Especially, two DEGs encoding the heat shock 70 kDa protein (HSP70) were significantly down-regulated. The HSP70 is one of the most prominent stress proteins and can be induced by a variety of stresses, such as UV radiation, heat shock, hypertonicity and low temperature (Tavaria et al., 1996; Alfieri et al., 2002; Fulgentini et al., 2015). This result showed that the deregulation of genes associated with the stress tolerance and especially the down-regulation of the genes encoding the HSP70, probably causing the suppression of stress tolerance of *P. infestans* under the melatonin treatment.

**Table 6:** The differentially expressed genes related to stress in transcriptome.

**Gene_id**	**Log2Foldchange**	**Padj**	**Interpro description**
PITG_05579	0.67299	0.024625	Catalase-peroxidase, putative
PITG_10772	0.47203	0.026249	Putative GPI-anchored elicitin INL11b-like protein
PITG_12561	0.8956	0.008554	Elicitin INF2A-like protein
PITG_12555	1.0902	7.53E-07	Elicitin-like protein, putative
PITG_06373	0.93318	0.001776	Putative GPI-anchored serine-rich elicitin INL3b-like protein
PITG_16907	1.5662	4.22E-20	Elicitin-like protein
PITG_10284	−1.3374	7.98E-11	Cellulose binding elicitor lectin (CBEL), putative
PITG_11249	−0.90696	7.63E-08	Heat shock 70 kDa protein
PITG_05498	−0.52905	0.005572	Heat shock protein 90, putative
PITG_13826	−0.45753	0.02728	Hybrid signal transduction histidine kinase, putative
PITG_20715	−0.52667	0.008664	Deoxyribodipyrimidine photolyase, putative
PITG_11247	−0.72089	7.62E-05	Heat shock 70 kDa protein
PITG_02805	−0.73632	3.88E-05	DNA mismatch repair protein Msh2, putative
PITG_22799	−1.1375	0.006449	Elicitin-like protein INL3B
PITG_04709	−0.43251	0.043581	DNA ligase

### Differentially expressed genes related to drug resistance of *P. infestans* were affected by melatonin

The transcriptome data also showed that the expression of some DEGs related to drug resistance was also affected by melatonin. The down-regulation of drug resistance genes by melatonin treatment was possibly the reason for the synergistic effects with Infinito. For example, among the 25 most down-regulated differentially expressed genes, there were two genes encoding major facilitator superfamilies (MFS) (Figure S3). MFS is regarded as a major mechanism of drug resistance by transporting the drug from the cytoplasm to extracellular space (Kumar et al., 2013). The overexpression of MFS can confer the cell with resistant to many toxic compounds, while the mutant of MFS cannot (Morschhauser, 2010; Kumar et al., 2013). These results suggested that the down-regulation of MFS gene expression could lead to the reduction of the drug resistance of *P. infestans*. Thus, the down-regulated MFS in transcriptome could weaken the drug resistance of *P. infestans*, leading to the synergic effects of melatonin combination with Infinito, such as reducing the use and increasing cytotoxicity of Infinito. Furthermore, consistent with the previous study, melatonin is able to overcome the clofarabine resistance in leukemic cell lines: combination of melatonin and clofarabine significantly increase cytotoxicity in resistant leukemic cell lines (Yamanishi et al., 2015).

### Differentially expressed genes related to virulence of *P. infestans* were affected by melatonin

The transcriptome data also showed that the expression of some DEGs related to virulence was also affected by melatonin. *P. infestans*, like other pathogens, can employ proteins related to virulence to facilitate colonization and infection, such as the apoplastic effectors which are secreted into the plant extracellular, cytoplasmic effectors which are translocated into the plant cell, Cytochrome P450, (Haas et al., 2009; Tian et al., 2009; Dong et al., 2014). Elicitins are apoplastic effectors for helping pathogen invasion into plants in a negative manner (Schornack et al., 2009). The reduced expression of elicitins help the pathogen to evade the host innate immunity, and the mutations of elicitin INF1 of *P. infestans* could cause the disease lesions (Kamoun et al., 1998; Schornack et al., 2009). Our transcriptome data showed that six genes encoding elicitin proteins were up-regulated but two were down-regulated in response to melatonin treatment (Table 7), suggesting that the virulence of *P. infestans* was reduced by melatonin. Most of these virulence-related proteins likely help pathogen to more access and entry into plants and in causing serious disease, by higher expression, such as: CBEL (apoplastic effector), Crinkler protein (CRN) (more than 200) (cytoplasmic effectors), and many Cytochrome P450 (Beyer et al., 2001; Gaulin et al., 2002; Haas et al., 2009). For example, the CRN protein mutant reduced the virulence of pathogen (Amaro et al., 2017). The down-regulated genes encoding CRN, CBEL, and Cytochrome P450 were major in the transcriptome data (Table 7), indicating that the virulence of *P. infestans* was impaired by melatonin. Thus, deregulation of genes by melatonin associated with the virulence might reduce the virulence of the pathogen invading the host, which could be one of the reasons for the attenuation of potato late blight in our observations.

**Table 7:** The differentially expressed genes related to pathogenesis in transcriptome.

**Gene_id**	**Log2Foldchange**	**Padj**	**Interpro description**
PITG_01370	−0.6879	0.000424	ATP-binding Cassette (ABC) Superfamily
PITG_05987	1.199	3.91E-07	ATP-binding Cassette (ABC) superfamily
PITG_06852	−0.73087	2.83E-05	ATP-binding Cassette (ABC) Superfamily
PITG_06860	1.2086	0.004669	ATP-binding Cassette (ABC) Superfamily
PITG_06862	0.99317	0.01237	ATP-binding Cassette (ABC) Superfamily
PITG_06863	1.038	3.53E-10	ATP-binding Cassette (ABC) Superfamily
PITG_07134	0.66538	0.000928	ATP-binding Cassette (ABC) Superfamily
PITG_07716	0.60179	0.00174	ATP-binding Cassette (ABC) Superfamily
PITG_07717	0.53475	0.00836	ATP-binding Cassette (ABC) Superfamily
PITG_11373	−0.56642	0.025893	ATP-binding Cassette (ABC) superfamily
PITG_13282	0.58644	0.007202	ATP-binding Cassette (ABC) Superfamily
PITG_13558	−0.6312	0.000553	ATP-binding Cassette (ABC) Superfamily
PITG_13575	0.48026	0.021395	ATP-binding Cassette (ABC) Superfamily
PITG_15731	0.56494	0.009076	ATP-binding Cassette (ABC) Superfamily
PITG_17053	0.8252	1.21E-05	ATP-binding Cassette (ABC) Superfamily
PITG_20815	1.5519	2.82E-21	ATP-binding Cassette (ABC) Superfamily
PITG_04809	−0.62118	0.006352	Crinkler (CRN) domain, pseudogene
PITG_14319	−4.8844	0.008491	Crinkler (CRN) family protein
PITG_04435	−0.54746	0.030686	Crinkler (CRN) family protein
PITG_22856	−0.66451	0.001265	Crinkler (CRN) family protein
PITG_23273	−1.3231	3.6E-06	Crinkler (CRN) family protein
PITG_14343	−0.66068	0.028973	Crinkler (CRN) family protein
PITG_16584	−0.45693	0.037047	Crinkler (CRN) family protein
PITG_16614	−0.73791	0.042338	Crinkler (CRN) family protein
PITG_16619	−0.79746	0.000581	Crinkler (CRN) family protein
PITG_18503	−0.63181	0.010154	Crinkler (CRN) family protein
PITG_19589	−0.68915	0.000276	Crinkler (CRN) family protein
PITG_20171	−0.63579	0.00405	Crinkler (CRN) family protein
PITG_22186	−0.63536	0.034212	Crinkler (CRN) family protein, pseudogene
PITG_03400	−0.51195	0.017922	Crinkler (CRN) family protein, putative
PITG_04768	−0.52386	0.048344	Crinkler (CRN) family protein, putative
PITG_17517	−0.70149	0.000199	Crinkler (CRN) family protein, putative
PITG_07699	−1.3195	6.58E-15	Cytochrome P450, putative
PITG_14013	−0.74111	3.33E-05	Cytochrome P450, putative
PITG_14388	−0.58475	0.029546	Cytochrome P450, putative
PITG_21524	0.95041	1.23E-06	Cytochrome P450, putative
PITG_12561	0.8956	0.008554	Elicitin INF2A-like protein
PITG_16907	1.5662	4.22E-20	Elicitin-like protein
PITG_20414	−0.66577	0.001878	Elicitin-like protein
PITG_22799	−1.1375	0.006449	Elicitin-like protein INL3B
PITG_12555	1.0902	7.53E-07	Elicitin-like protein, putative
PITG_10772	0.47203	0.026249	Putative GPI-anchored elicitin INL11b-like protein
PITG_20412	0.53758	0.010979	Putative GPI-anchored serine rich elicitin SOL13E-like protein
PITG_06373	0.93318	0.001776	Putative GPI-anchored serine-rich elicitin INL3b-like protein
PITG_02830	−2.3632	6.03E-28	Secreted RxLR effector peptide protein, putative
PITG_15235	−2.1923	5.28E-17	Secreted RxLR effector peptide protein, putative
PITG_22675	0.983	0.012687	Secreted RxLR effector peptide protein, putative
PITG_03192	2.5813	8.22E-05	Secreted RxLR effector peptide protein, putative
PITG_04049	1.1756	1.45E-09	Secreted RxLR effector peptide protein, putative
PITG_06308	1.4386	3.75E-08	Secreted RxLR effector peptide protein, putative
PITG_10396	−1.0182	0.032769	Secreted RxLR effector peptide protein, putative
PITG_10654	−0.75283	0.010483	Secreted RxLR effector peptide protein, putative
PITG_11947	−0.59988	0.040855	Secreted RxLR effector peptide protein, putative
PITG_13847	0.55452	0.024395	Secreted RxLR effector peptide protein, putative
PITG_14884	1.0544	0.000997	Secreted RxLR effector peptide protein, putative
PITG_23035	0.65992	0.001252	Secreted RxLR effector peptide protein, putative
PITG_15039	0.76481	0.007932	Secreted RxLR effector peptide protein, putative
PITG_15125	0.89507	0.00824	Secreted RxLR effector peptide protein, putative
PITG_16427	0.97811	1.08E-05	Secreted RxLR effector peptide protein, putative
PITG_23092	2.6575	2.54E-11	Secreted RxLR effector peptide protein, putative
PITG_17309	0.91868	1.44E-05	Secreted RxLR effector peptide protein, putative
PITG_17316	1.014	7.82E-06	Secreted RxLR effector peptide protein, putative
PITG_04085	0.6237	0.006739	Avrblb2 family secreted RxLR effector peptide protein, putativeAvrblb2 family secreted RxLR effector peptide, putative
PITG_04086	0.69051	0.001675	Avrblb2 family secreted RxLR effector peptide protein, putativeAvrblb2 family secreted RxLR effector peptide, putative
PITG_20300	0.92685	0.002562	Avrblb2 family secreted RxLR effector peptide, putative
PITG_10284	−1.3374	7.98E-11	Cellulose binding elicitor lectin (CBEL), putative

### Differentially expressed genes related to circadian rhythms of *P. infestans* treated by melatonin were not observed

The function of melatonin is well known to regulate circadian rhythms. The circadian rhythms also may regulate the physiological function of fungus, including virulence and mycelia (Liu and Bellpedersen, 2006; Brody et al., 2010; Hevia et al., 2015). Therefore, we investigated whether melatonin altered circadian rhythms-related genes of *P. infestans*. However, the core circadian rhythms genes of fungus, such as FRQ, WC-1,WC-2 (Liu and Bellpedersen, 2006; Salichos and Rokas, 2010), were not observed in the transcriptome data; the GO terms or KEGG pathway involved in circadian rhythms were also not detected in transcriptome data (Tables S4, S5). This observation likely caused by the continuous darkness growth condition of *P. infestans* in this study.

### Validation of gene expression profiles using RT-qPCR

To confirm these transcriptome-based observations, nine DEGs encoding rate-limiting enzymes in metabolism, and four DEGs involved in pathogenesis were selected for the real-time PCR (RT-qPCR) based confirmation studies. The results were highly consistent with these in the in transcriptome dataset (Figure S4), suggesting that the datasets derived from the transcriptome assay are reliable and valid.

## Conclusion

In this study, exogenous application of melatonin significantly attenuated the potato late blight by inhibiting mycelial growth, changing cell ultrastructure, reducing the virulence and impairing stress tolerance of *P. infestans*. Importantly, melatonin reduced the doses and enhanced the effects of fungicide for potato late blight control, because of the synergistic inhibitory effects generated by co-treating with melatonin and fungicide. The analysis of the transcriptome suggests that the inhibitory effects of melatonin on *P. infestans* is most likely due to altering the homeostasis of amino acid metabolism. Furthermore, melatonin also altered the expression of important factors associated to stress tolerance, fungicide resistance and virulence. These observations also revealed the direct inhibitory effects of melatonin on the growth of *P. infestans* for the first time and provided new insights into the mechanisms of the direct interaction between melatonin and plant pathogens. Melatonin treatment showed some advantages in for the control of potato late blight in comparison with traditional chemical fungicides and potential application valuable in the cultivation of resistant variety. Firstly, the commonly used chemical fungicides usually display toxicity to humans and animals, whereas melatonin shows less toxicity even under high concentrations. Secondly, as a potential synergist, melatonin has great potential to reduce the use of chemical fungicides. It not only maximizes the efficiency of chemical fungicides and minimizes the use of chemical fungicides, but also reduces fungicide pollution. Additionally, there is potential to develop novel transgenic potato plants which may not only produce more melatonin but also generate the small RNA targeting the key regulators of amino acid metabolism in *P. infestans*, by manipulating the key genes controlling the biogenesis of melatonin in potato and amino acid metabolism in *P. infestans*, respectively.

## Author contributions

MR and SZ designed the research; SZ, XZ, SF, SL, LJ, and YW performed research; MR, SZ, RR, ZL, and RD analyzed data; MR, SZ, RR wrote the paper.

### Conflict of interest statement

The authors declare that the research was conducted in the absence of any commercial or financial relationships that could be construed as a potential conflict of interest.

## Supplementary material

Figure S1 **(A)** The lesion size of leaves infected by *P. infestans* treated with DMSO, melatonin, Infinito, melatonin + Infinito, respectively, after 5 days. **(B)** The lesion size of tuber slices treated with DMSO, melatonin, Infinito, melatonin + Infinito, respectively, after 5 days.

Figure S2 The detail heat-map of total DEGs induced by melatonin.

Figure S3 The top 25 down-regulated genes. 1. Putative uncharacterized protein; 2. Hypothetical protein O9G_005006; 3. Unknown protein; 4.Crinkler (CRN) family protein; 5. Unknown protein; 6. Unknown protein; 7. 12-oxophytodienoate reductase, putative; 8. Putative uncharacterized protein; 9. Putative uncharacterized protein; 10. Unknown protein; 11. Alcohol dehydrogenase, putative; 12. Major Facilitator Superfamily (MFS); 13. Putative uncharacterized protein; 14. Unknown protein; 15. Alcohol dehydrogenase, putative; 16. Secreted RxLR effector peptide protein, putative; 17. Putative uncharacterized protein; 18. Putative uncharacterized protein; 19. Putative uncharacterized protein; 20. Secreted RxLR effector peptide protein, putative; 21. Aquaporin, putative; 22. Major Facilitator Superfamily (MFS); 23. Putative uncharacterized protein; 24. Putative uncharacterized protein; 25. Putative uncharacterized protein.

Figure S4 Expression levels of 9 genes encoding rate-limiting enzymes and 4 genes related to pathogenesis in the 3 mM melatonin treatment for 24 h. 1. 6-phosphofructokinase, putative; 2. Pyruvate kinase; 3. Fructose-bisphosphate aldolase; 4. Glycerol-3-phosphate O-acyltransferase, putative; 5. Isocitrate dehydrogenase [NADP]; 6. Aconitate hydratase, putative; 7. Acetyl-CoA carboxylase, putative; 8. Glucokinase, putative; 9. Glucokinase, putative; 10. Crinkler (CRN) family protein, putative; 11. Cytochrome P450, putative; 12. Cellulose binding elicitor lectin (CBEL), putative; 13. Elicitin INF2A-like protein.
