# Supplementary figures and images for: Melatonin Attenuates Potato Late Blight by Disrupting Cell Growth, Stress Tolerance, Fungicide Susceptibility and Homeostasis of Gene Expression in Phytophthora infestans
Source: Front Plant Sci. 2017 Nov 21;8:1993. doi: 10.3389/fpls.2017.01993 (PMC5702310; doi:10.3389/fpls.2017.01993)

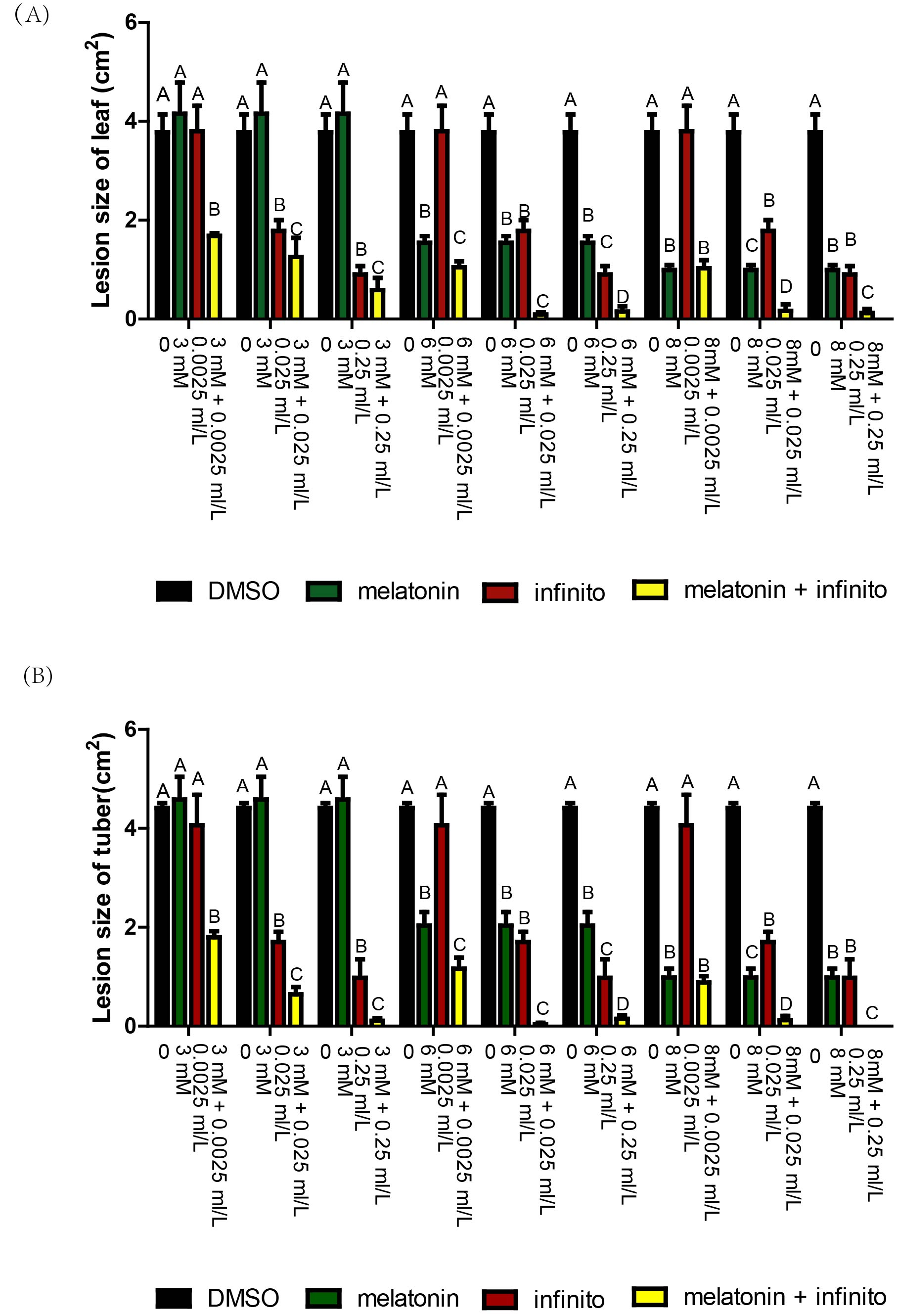

Supplement: Figure S1 — (A) The lesion size of leaves infected by P. infestans treated with DMSO, melatonin, Infinito, melatonin + Infinito, respectively, after 5 days. (B) The lesion size of tuber slices treated with DMSO, melatonin, Infinito, melatonin + Infinito, respectively, after 5 days. [file Image1.JPEG]

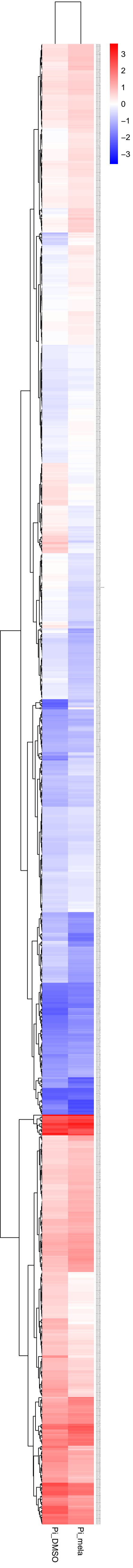

Supplement: Figure S2 — The detail heat-map of total DEGs induced by melatonin. [file Image2.PDF]

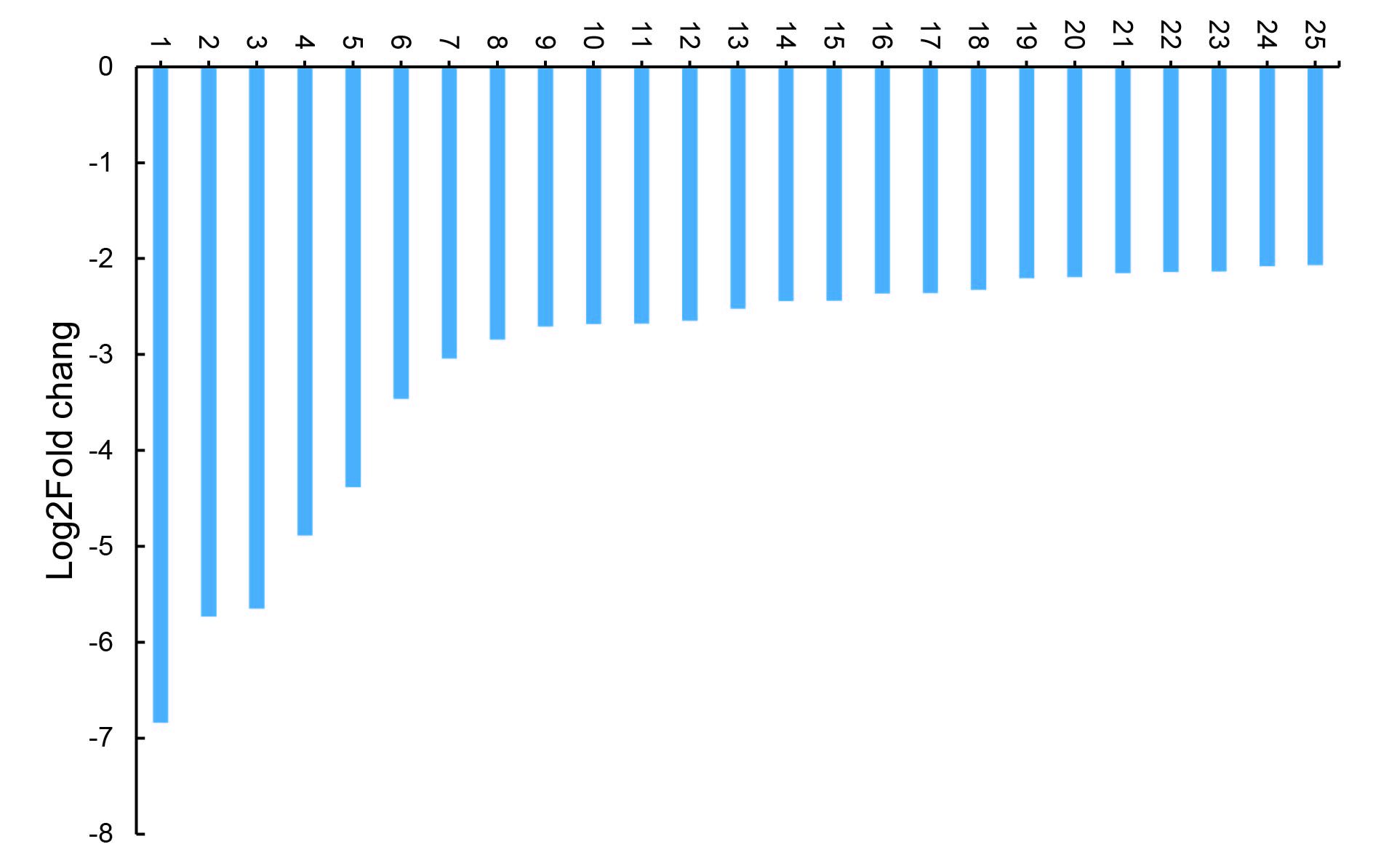

Supplement: Figure S3 — The top 25 down-regulated genes. 1. Putative uncharacterized protein; 2. Hypothetical protein O9G_005006; 3. Unknown protein; 4.Crinkler (CRN) family protein; 5. Unknown protein; 6. Unknown protein; 7. 12-oxophytodienoate reductase, putative; 8. Putative uncharacterized protein; 9. Putative uncharacterized protein; 10. Unknown protein; 11. Alcohol dehydrogenase, putative; 12. Major Facilitator Superfamily (MFS); 13. Putative uncharacterized protein; 14. Unknown protein; 15. Alcohol dehydrogenase, putative; 16. Secreted RxLR effector peptide protein, putative; 17. Putative uncharacterized protein; 18. Putative uncharacterized protein; 19. Putative uncharacterized protein; 20. Secreted RxLR effector peptide protein, putative; 21. Aquaporin, putative; 22. Major Facilitator Superfamily (MFS); 23. Putative uncharacterized protein; 24. Putative uncharacterized protein; 25. Putative uncharacterized protein. [file Image3.JPEG]

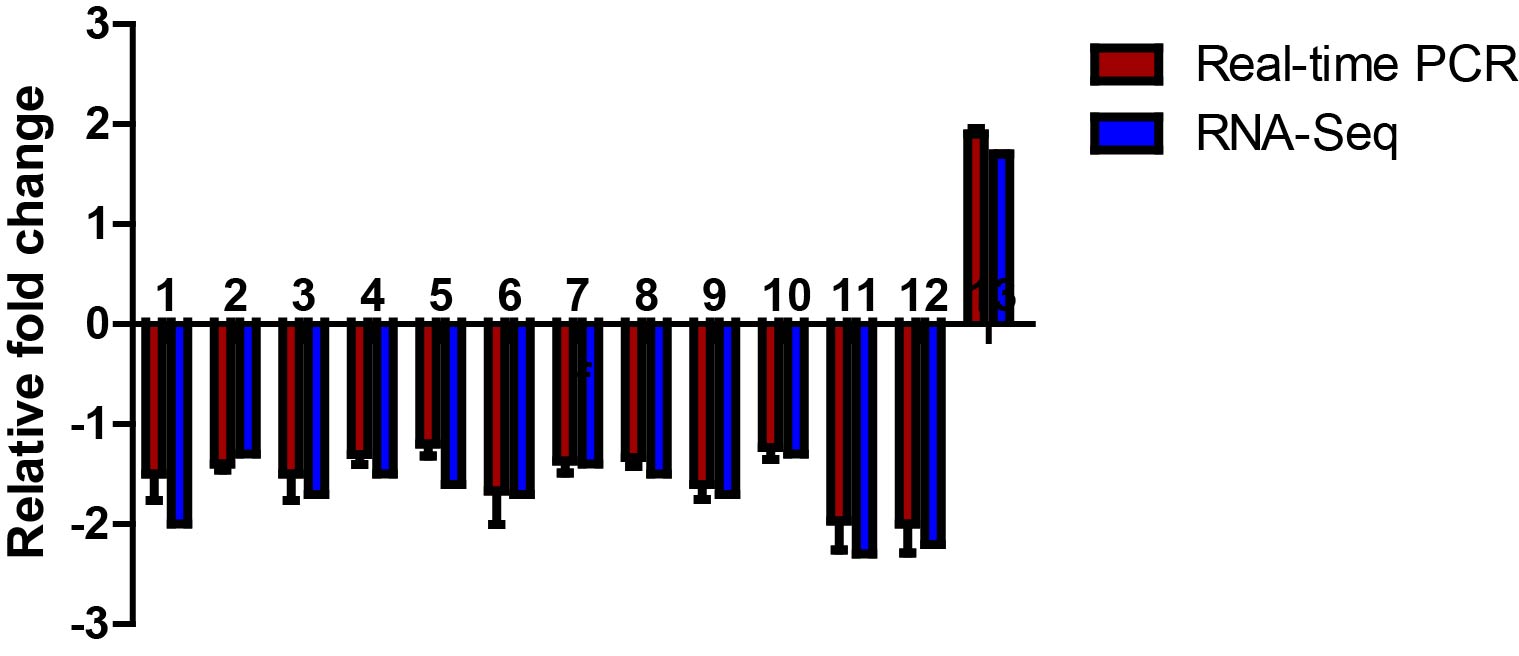

Supplement: Figure S4 — Expression levels of 9 genes encoding rate-limiting enzymes and 4 genes related to pathogenesis in the 3 mM melatonin treatment for 24 h. 1. 6-phosphofructokinase, putative; 2. Pyruvate kinase; 3. Fructose-bisphosphate aldolase; 4. Glycerol-3-phosphate O-acyltransferase, putative; 5. Isocitrate dehydrogenase [NADP]; 6. Aconitate hydratase, putative; 7. Acetyl-CoA carboxylase, putative; 8. Glucokinase, putative; 9. Glucokinase, putative; 10. Crinkler (CRN) family protein, putative; 11. Cytochrome P450, putative; 12. Cellulose binding elicitor lectin (CBEL), putative; 13. Elicitin INF2A-like protein. [file Image4.JPEG]
